# The efficacy of steroids in reducing morbidity and mortality from extreme hyperthermia and heatstroke—A systematic review

**DOI:** 10.1002/prp2.626

**Published:** 2020-07-14

**Authors:** Edward Walter, Oliver R. Gibson

**Affiliations:** ^1^ Intensive Care Unit Royal Surrey County Hospital Guildford UK; ^2^ Division of Sport, Health and Exercise Sciences Centre for Human Performance, Exercise and Rehabilitation (CHPER) Brunel University London Uxbridge UK

**Keywords:** heat stress, hyperthermia, outcomes, steroids

## Abstract

Severe hyperthermia from classical or exertional heatstroke, or from drug ingestion or other noninfective pyrogens, is associated with a high mortality and morbidity. A systemic pro‐inflammatory response occurs during heatstroke, characterized by elevated cytokines with endotoxemia from elevated lipopolysaccharide (LPS) levels. Corticosteroids reduce LPS and cytokine levels, suggesting that they may improve outcome. A systematic review searching Embase, MEDLINE, and PubMed from the earliest date available until September 2019 was conducted, according to the PRISMA guidelines, with five papers identified. In four studies, systemic steroids administered before or at the onset of heat stress improved mortality or reduced organ dysfunction. Survival time was greatest when steroid administration preceded heat stress. In one study, a nonsignificant increase in mortality was seen. A dose response was observed, with higher doses extending survival time. Animal studies suggest that steroids improve mortality and/or organ dysfunction after an episode of heat stress or extreme hyperthermia.

**Abbreviations**
AKI: acute kidney injury
CBF: cerebral blood flow
CHS: classical heatstroke
EHS: exertional heatstroke
GI: gastrointestinal
IL‐1: interleukin‐1
IL‐10: interleukin‐10
IL‐1β: interleukin‐1β
IL‐6: interleukin‐6
INFγ: interferon gamma
LPS: lipopolysaccharide
MAP: mean arterial pressure
NMS: neuroleptic malignant syndrome
T_CORE_: core temperature
TNF‐α: tumor necrosis factor‐alpha

## 1 INTRODUCTION

Hyperthermia induces deleterious effects at the cellular, organ, and whole‐body level,^1^ and has a variety of causes, which include sepsis, classical and exertional heat illness, and drug‐induced hyperthermia. While there is a survival benefit to a mild pyrexia in sepsis,^2^, ^3^ mortality increases as the core temperature (*T*_CORE_) exceeds 40°C,^2^ suggesting that at higher temperatures, the deleterious effects on organ and cellular function outweigh any benefit conferred from pyrexia. In noninfective hyperthermia, however, a *T*_CORE_ of 38.5°C or greater, is associated with a worse outcome.^3^

Heatstroke represents the most severe form of heat illness with significant morbidity and mortality, including long‐term multiorgan dysfunction and susceptibility to further heat illness. Exertional heatstroke (EHS) occurs in individuals undergoing strenuous physical activity, especially in hot and humid conditions, and is defined as a *T*_CORE_ above 40.5°C with neurological dysfunction. It is the third commonest cause of death in athletes.^4^ Classical heatstroke (CHS) presents with similar symptoms to EHS but in the absence of exercise or exertion. CHS is often seen in meteorological heat waves and has a 28‐day mortality rate of 58%, increasing to 71% at 2 years.^5^ The clinical, biochemical, and physiological similarities between other noninfective hyperthermic states, for example, after drug ingestion, suggest that the pathological changes are at least partly due to hyperthermia per se, irrespective of the cause. A large number of drugs appear to have hyperthermic properties^6^, ^7^; common categories include serotonergic (eg, antidepressant agents and opioids), anticholinergic (eg, antihistamine and antipsychotic agents), volatile anesthetic agents, and neuroleptic medications. A number of mechanisms are thought to be responsible for the hyperthermia, including reduction in heat dissipation, and changes in uncoupling protein function^8^; the latter appears to allow diversion of protons normally involved in the production of ATP to instead be dissipated as heat.^9^

Current treatment options for heatstroke and severe hyperthermia remain limited. The priority remains rapid cooling, to a *T*_CORE_ below 38.6°C, ideally at a rate greater than 0.16°C·min^−1^,^10^, ^11^ and supportive treatment. While a specific treatment may be efficacious in a particular condition, (eg, dantrolene in malignant hyperthermia, and cyproheptadine in serotonin syndrome), drug treatment is not generally recommended or of benefit in most cases.^6^, ^11^ Hyperthermia is associated with the development of organ dysfunction (see below), which may require supportive treatment.

Hyperthermia is directly cytotoxic, affecting membrane stability and transmembrane transport protein function with electrolyte homeostasis, protein, and DNA synthesis disrupted. The nuclear matrix shows damage at lower temperatures than other parts of the cell, with significant endothermic changes observed at 40°C.^12^ Direct cell death in humans occurs at temperatures of around 41°C, with the rate of cell death increasing markedly with further temperature increases, primarily due to protein denaturation.^13^, ^14^ The microvasculature is affected rapidly during hyperthermia, with capillary dilation, vascular stasis, and extravasation into the interstitium at a temperature of 40.5°C.^15^ Renal glomerular filtration rate reduces after a *T*_CORE_ increase of 2°C and worsens further with increasing temperature.^16^ Acute kidney injury (AKI) affects one in six hospitalized patients with EHS^17^ and has been reported in 53% after CHS.^18^ Renal failure sufficient to require renal replacement therapy has also been described after hyperthermia due to neuroleptic malignant syndrome,^19^ malignant hyperthermia,^20^ and recreational drug use.^21^ Hepatocellular dysfunction is common at temperatures above 40°C,^22^ and coagulopathy occurs with a reported incidence of 45% in CHS.^18^ The integrity of the blood‐brain barrier is disrupted,^23^ allowing translocation of systemic toxins into the cerebral circulation.

The gastrointestinal (GI) barrier is composed of physical factors such as enterocyte membranes and tight junctions between enterocytes, along with an immunological defense system, to minimize translocation of toxic substances from the intestinal lumen to the internal environment. Systemic hyperthermia increases the permeability of the gastrointestinal tract increasing the rate of gut bacterial translocation. Exposure leading to a *T*_CORE_ exceeding 41.6°C‐42.0°C, even after 60 minutes, induces a rapid sloughing of intestinal epithelial surface and an increase in intestinal permeability, including to large molecules up to a molecular weight of 4000 Da.^24^ More modest increases in temperature to 39°C‐41°C have been shown in vitro to cause an increase in paracellular permeability.^25^ The changes to permeability happen early, within a few hours, but are reversible—paracellular permeability returns to normal even if the hyperthermia is maintained for 24 hours^25^ In addition to these direct effects of hyperthermia, blood flow to the intestinal tract is reduced at temperatures above 40°C.^26^ This exacerbates the loss of the GI barrier integrity and increases the potential for endotoxemia and systemic lipopolysaccharide (LPS) increases, which initiates the release of pro‐inflammatory cytokines.^27^, ^28^

Lipopolysaccharides are large molecules forming part of the outer membrane of gram‐negative bacteria. LPS contains a hydrophobic domain, known as endotoxin,^29^ which stimulates release of pro‐inflammatory mediators if they enter the systemic circulation. A pro‐inflammatory response is a well‐developed defense mechanism, triggered by infective pathogens and toxic insults, such as trauma, and removes injurious stimuli and initiates tissue repair. Pro‐inflammatory cytokines, for example, tumor necrosis factor‐alpha (TNF‐α) and interleukin‐1 (IL‐1), are released primarily from macrophages and mast cells; cytokines and cell migration to the site of injury promote neutralization of the antigen and tissue repair and healing. In one study, LPS was elevated in 100% patients admitted to hospital after heatstroke, coinciding with a *T*_CORE_ of 42.1°C.^30^ Significantly increased LPS levels have also been found in the portal circulation of heat‐stressed rats at a *T*_CORE_ of 41.5°C.^26^ Heat stress in primates increases portal and systemic LPS concentration.^31^ LPS is thought to be responsible for some of the deleterious effects of hyperthermia—administration of purified LPS produces diffuse endothelial injury, tissue hypoperfusion, and refractory shock,^28^ and attenuation of systemic LPS by anti‐LPS antibodies^32^ improves survival after heat stress. Administration of antibiotics against intestinal microorganisms prevents endotoxemia from occurring^33^ and appears to improve mortality,^34^, ^35^ suggesting that endotoxemia occurring with heat stress is gut derived. Endurance exercise also produces endotoxemia,^36^, ^37^ but whether this is primarily related to *T*_CORE_ increases is not certain. In one cross‐over study, an increase in the intensity of exercise increased the *T*_CORE_ and intestinal permeability,^38^ and the cytokine profile of EHS and CHS is similar to that produced in endotoxemia,^39^ suggesting that irrespective of the initial stress, the resulting gut‐derived endotoxemia may be similar. Abolition of endotoxemia significantly reduces cytokine production.^39^

The role of cytokines in heat stress is unclear with an inconsistent response to thermal stress. A number of pro‐inflammatory and anti‐inflammatory cytokines are elevated at the time of hyperthermia from heatstroke. Acute phase reactants may also increase. Of these, some (eg, interferon gamma [INFγ], interleukin‐1β [IL‐1β]) are raised in a proportion of patients, whereas interleukin‐6 (IL‐6) may be elevated in all patients.^40^ There is some correlation with outcome; the rise in IL‐6 and the duration of the increased expression is related to mortality, independent of the maximum core temperature obtained.^41^ Mice pretreated with IL‐6 before exposure to heat take longer to reach a *T*_CORE_ of 42.4°C, showing less organ damage, and attenuation in the increase of other cytokines.^42^ Antagonism of IL‐1 also improves survival.^43^ Development of other hyperthermic states may also be associated with inflammatory mediators. Neuroleptic malignant syndrome (NMS) may be at least partly driven by an acute phase response; acute phase response mediators are reported to rise, and peak at 72 hours. Conversely, levels of anti‐inflammatory agents such as serum iron and albumin initially decline then return to the normal range, coinciding with clinical improvement.^44^ IL‐6 and TNF‐α levels have also been found to be significantly increased in NMS,^45^ and IL‐6 in malignant hyperthermia.^46^

Glucocorticoids inhibit many of the initial events in an inflammatory response, promoting the resolution of inflammation.^47^ Acutely, glucocorticoids inhibit the vasodilation and increased vascular permeability that occurs following an inflammatory insult and decrease leukocyte migration to the site of injury.^48^ Most of the anti‐inflammatory and immunosuppressive actions of glucocorticoids are attributable to alterations in the genetic transcription in leukocytes.^48^ While the precise role of the inflammatory response in heatstroke is unclear, reducing the inflammation appears to be of benefit. Corticosteroids reduce levels of the majority of cytokines,^47^ and the administration of prophylactic glucocorticoids prevents heatstroke‐induced LPS rise in an animal model.^49^, ^50^ These data therefore suggest that administration of corticosteroids may have a beneficial role in the treatment of heatstroke.

## 2 METHODS

Evidence for the clinical effectiveness of steroids in the acute treatment of hyperthermia and heatstroke was assessed by conducting a systematic review of published research evidence. The review adhered to the PRISMA checklist (Appendix A).^51^

### 2.1 Identification of studies

RCTs were identified by searching three electronic medical databases (Embase, MEDLINE, and PubMed), from the earliest date until September 2019. In addition, the EU Clinical Trials Register and the Cochrane library were searched. Further attempts to identify studies were made by examining the reference lists of all retrieved articles and review articles identified by the original searches. “Cancer” terms were excluded from searches, to exclude the studies on hyperthermia as a treatment modality.

The search terms used for the three searches are summarized as follows, and detailed in Appendix B. The Embase database was searched from 1974 to September 2019, using the “explosion” search terms of “heat stress,” “heat injury,” “hyperthermia,” and “steroid.” The MEDLINE database was searched from 1950 to August 2019, using the “explosion” search terms of “heat stress disorders” and “steroids.” The PubMed database was searched from 1966 to August 2019, using the search terms “steroid*,” “heat illness,” “heat stroke,” and “heat stress” in the titles or abstracts. No limits on any searches were set.

### 2.2 Inclusion and exclusion criteria

Two reviewers independently screened all titles and abstracts. Full‐text papers of any titles and abstracts that were considered relevant by either reviewer were obtained where possible. The relevance of each study was assessed according to the inclusion criteria stated in Table 1. Studies that did not meet the criteria were excluded. Any discrepancies were resolved by consensus.

**TABLE 1:** Study design and characteristics to be identified from the search

Study design	RCTs
Population(s)	Animal or human studies
Intervention(s)	Administration of steroid before or after exposure to hyperthermia or heat stress
Comparators	The intervention will be compared with the control group
Outcomes	Survival or organ dysfunction

### 2.3 Types of intervention

Published randomized controlled trials (RCTs) where steroids were administered to animals or humans before or after the onset of heat stress, compared against placebo, were sought.

### 2.4 Outcome measures

Trials where the steroids were assessed against survival data or evidence of organ dysfunction were included. These outcomes represent the most clinically relevant responses. Survival data were taken as the primary outcome; organ dysfunction as the secondary outcome.

### 2.5 Assessment of bias

For preclinical or animal studies, the reports were assessed for bias by using the Syrcle risk of bias tool.^52^

### 2.6 Measures of treatment effect

We included any measure of mortality, including survival time, temperature at which death occurred, and absolute survival numbers after cessation of heat insult or trial. A *P* value of less than .05 was taken as statistically significant.

### 2.7 Exclusion criteria

Studies were excluded in part or in total if the steroids were given in combination with another treatment. Results were only included if the effect of steroids alone was compared with the control group, either in a subgroup or the study as a whole.

### 2.8 Subgroup analysis

Additional statistical analysis between the intervention and control groups was undertaken where appropriate if not reported in the study. Statistical analysis performed by the review authors is highlighted in the text; all other analyses were extracted from the study. The Student's *t* test was used for continuous outcome data; the chi‐squared statistic for discrete outcome data.

## 3 RESULTS

Electronic searches identified 8553 citations. Hand searches revealed no further studies. Titles and abstracts were assessed for relevance to the review (stage 1 screening), and duplications were identified, resulting in 63 potential citations being retained. The full texts of these citations were obtained. After applying inclusion criteria to these full‐text papers (stage 2 selection), 58 citations, which did not meet the inclusion criteria, were excluded. Five citations were therefore included in the systematic review (Appendix C). No studies were found that investigated the secondary outcome that did not also investigate mortality.

Five studies were found which met the criteria (Tables 2 and 3). Of the five studies, three used rats^53^, ^54^, ^55^ and two used primates.^50^, ^56^ No human studies were found. Four of the studies used dexamethasone^53^, ^54^, ^55^, ^56^ and one methylprednisolone.^50^ In three studies, the steroid was given after the onset of heat stress.^53^, ^54^, ^55^ With the exception of one study,^56^ all studies reported improved survival (three reaching statistical significance) and markers of organ dysfunction.

**TABLE 2:** Study characteristics

Study	Date of study	Country	Commercial/financial support
Lui	2000	Taiwan	National Science Council of the Republic of China Veterans’ General Hospital‐National Yang‐Ming University joint research program Tsou's Foundation Ministry of Education of the Republic of China
Lui	2014	Taiwan	National Science Council of the Republic of China
Bouchama	2007	Saudi Arabia	King Faisal Specialist Hospital & Research Center, Riyadh, Saudi Arabia.
Yang	2010	Taiwan	National Science Council of the Republic of China
Gathiram	1998	South Africa	Chamber of Mines, Johannesberg, SA

**TABLE 3:** Study results (* statistically different to control; ns = not significant (*P* > .05))

Study	Intervention	Number of subjects in intervention/control group	Species	Measure of mortality outcome	Summary of findings
Lui (2000)	4‐6 mg kg^−1^ dexamethasone (preinsult)	10/10	Rat	Time to death	101 ± 3 min (control) 250 ± 9 min (4 mg kg^−1^)* > 450 min (6 mg kg^−1^)*
	4‐6 mg kg^−1^ dexamethasone (onset of insult)	10/10		Time to death	100 ± 4 min (control) 122 ± 3 min (4 mg kg^−1^)* 321 ± 5 min (6 mg kg^−1^)*
Lui (2014)	4,6 or 8 mg kg^−1^ dexamethasone (onset of insult)	8 in each group	Rat	Survival time	24 ± 3 min (control) 104 ± 9 min (4 mg kg^−1^)* 204 ± 25 min (6 mg kg^−1^)* 268 ± 27 min (8 mg kg^−1^)*
Bouchama (2007)	2 mg kg^‐−1^ dexamethasone (immediately preinsult, and continuing during cooling)	5/5	Baboon	Time to death	10.9 ± 7.3 h (control) 11 ± 5.4 h (2 mg kg^−1^)(ns)
		5/5		Survival	3 (control) 2 (2 mg kg^−1^)(ns)
Yang (2010)	4 mg kg^−1^ dexamethasone (onset of insult)	8/8	Rats	Survival time	22 ± 3 min (control) 34 ± 6 min (4 mg kg^−1^)(ns)
Gathiram (1988)	30 mg kg^−1^ methylprednisolone (30m preinsult)	4/8	Monkeys	Temperature at death	44.9 ± 0.14°C (control) 44.4 ± 0.1°C (30 mg kg^−1^)*

**FIGURE 1:**
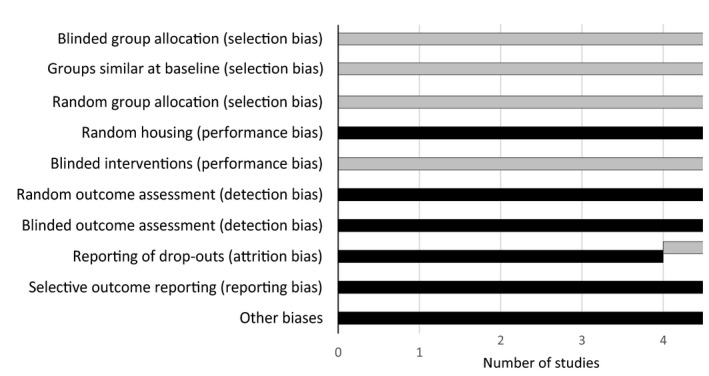
Risk of bias assessment, Key: Black—low risk of bias, Grey—unclear risk of bias, White—high risk of bias

All included studies were assessed for risk of bias (see Table 4 and Figure 1), and none were excluded after consideration of bias impact. All the papers stated that animals were allocated at random, but none described the allocation process in detail. None of the papers described or compared characteristics of the intervention and control groups separately or were randomly housed, but there was not any indication that there were differences between the groups. None of the papers stated that the investigators were blinded to the allocation, for example that the caregivers were separate to the investigators, but the reviewers considered that given that objective data were being collected in all cases, the reported outcomes are unlikely to have been affected by any lack of blinding. In four of the five papers, all study animals were accounted for, but in all papers, the results of all the proposed outcomes were reported.

**TABLE 4:** Risk of bias assessment

	Bouchama (2007)	Gathiram (1988)	Lui (2000)	Lui (2014)	Yang (2010)
1) Was the allocation sequence adequately generated and applied?	U	U	U	U	U
2) Were the groups similar at baseline or were they adjusted for confounders in the analysis?	U	U	U	U	U
3) Was the allocation to the different groups adequately concealed during?	U	U	U	U	U
4) Were the animals randomly housed during the experiment?	Y	Y	Y	Y	Y
5) Were the caregivers and/or investigators blinded from knowledge which intervention each animal received during the experiment?	U	U	U	U	U
6) Were animals selected at random for outcome assessment?	Y	Y	Y	Y	Y
7) Was the outcome assessor blinded?	Y	Y	Y	Y	Y
8) Were incomplete outcome data adequately addressed?	Y	Y	Y	U	Y
9) Are reports of the study free of selective outcome reporting?	Y	Y	Y	Y	Y
10) Was the study apparently free of other problems that could result in high risk of bias?	Y	Y	Y	Y	Y

Note

Key: Y: Yes (low risk of bias), N: No (high risk of bias), U: Unclear (unclear risk of bias)

The studies were considered too heterogeneous in their methodology and outcome measures to enable completion of a metanalysis. A descriptive summary was therefore completed.

### 3.1 Mortality

Administration of 4 mg kg^−1^ of dexamethasone to rats, either before or after the onset of heatstroke (defined as the time a decrease in peak mean arterial pressure (MAP) and cerebral blood flow (CBF) in the striatum occurred), improved survival time from 101 ± 3 minutes (control) to 250 ± 9 minutes and 122 ± 3 minutes, respectively.^53^ Administration of a higher dose (6 mg kg^‐1^) before or after heatstroke onset further improved survival time to greater than 450 minutes, and 321 ± 5 minutes, respectively. These data highlight that the administration of steroids prior to the onset of heatstroke lengthens survival time compared with administration after the onset, and that the higher dose (6 mg kg^−1^) had greater benefit than the lower dose (4 mg kg^−1^) (statistical analysis for this review). Bilateral adrenalectomy was performed in a further subgroup; MAP, CBF, and time to death were significantly lower in this group, but these changes were attenuated by dexamethasone.

A later study^54^ observed incremental doses of dexamethasone at the onset of heatstroke increased survival time to 104 ± 9 minutes (4 mg kg^−1^), 204 ± 25 minutes (6 mg kg^−1^), and 268 ± 27 minutes (8 mg kg^−1^) compared with untreated controls (24 ± 3 minutes) (statistical analysis for this review).

The third study to use rats^55^ administered 4 mg kg^‐1^ dexamethasone at the onset of heatstroke, defined in the same way as the studies by Lui.^53^, ^54^ Survival time showed a trend toward improvement from 22 ± 3 to 34 ± 6 minutes (*P* = .09). This study also administered mannitol, an osmotically active diuretic and anti‐inflammatory agent used after neurotrauma, to a further group which improved histological and biomarker assessment of neuronal damage, blood pressure, and survival time further. This subgroup was excluded from our analysis since it did not meet the inclusion criteria for the review. All three studies induced heat stress by heating the anesthetized animals in a heat chamber at 42°C or 43°C. Heatstroke occurred between 70 and 90 minutes later. The rise in temperature in the intervention group was not different to the control group.

In the first of two studies to use primates, Gathiram^50^ administered 30 mg kg^−1^ methylprednisolone to four anesthetized primates 30 minutes before the onset of heating, by use of a heat chamber at 41°C. The steroid‐treated animals succumbed at a significantly higher temperature (44.9 ± 0.1 vs 44.4 ± 0.1°C) compared to controls. The rate of rise in *T*_CORE_ and the time taken to reach heatstroke was not recorded. In the study by Bouchama,^56^ 10 baboons were assigned randomly to dexamethasone or control group. Dexamethasone (2 mg kg^−1^) was administered in four divided doses every 6 hours starting immediately before heat stress and continuing during cooling. The rate of heating, maximum temperature, and time above 40.4°C was not significantly different between the two groups. However, two animals (40%) in the control group survived, compared with only one in the steroid‐treated group (*P* > .05). This study therefore opposes the positive findings observed in rat models. The use of a lower total dose, alongside the divided administration over a prolonged postinsult period may have led to the null findings which contrast with positive outcomes in studies where a larger dose is administered prior to or immediately after the onset of hyperthermia.

### 3.2 Organ failure

#### 3.2.1 Cardiovascular

All studies documented the effect of steroid administration on mean arterial blood pressure (MAP). Three studies^53^, ^54^, ^55^ demonstrated that heat stress reduced MAP compared with nonheated controls; four studies^50^, ^53^, ^54^, ^56^ showed that this hypotension was improved at specific time points with steroid administration. One study^50^ showed an improvement in MAP at specific core temperatures, which became statistically significant above 42°C. The heart rate was recorded in one study,^50^ which was lower at all temperatures compared with untreated but heated controls. Of the three papers recording cerebral blood flow (CBF), two^53^, ^54^ document an improvement after the onset of heatstroke compared to untreated but heated controls.

#### 3.2.2 Neurological damage

Two studies investigated neuronal damage by histological changes against a previously defined score,^53^, ^55^ with significant worsening compared with unheated controls and an improvement in the steroid‐treated group in both groups, one reaching statistical significance.^53^ Cerebral levels of three biochemical markers of neuronal damage (glutamate, glycerol, and lactate/pyruvate ratio) were shown to increase with heat stress, and were reduced significantly in the steroid‐treated group^54^; this reduction in the heat stress‐induced increase in cerebral biomarkers was not, however, seen in the study by Yang.^55^ The difference in effect across studies may be explained by the dose difference: the study by Yang used a dose of 4 mg kg^−1^, compared with the higher dose (8 mg kg^−1^) in the study by Lui, where a difference was seen.

#### 3.2.3 Inflammatory cytokines

Administration of steroids in one study prevented a detectable increase in serum LPS,^50^ and in another caused an improvement in the heatstroke‐activated inflammatory response as demonstrated by an improvement in the reduction in complement C3 and C4 levels and the increase in IL‐6 during cooling.^56^ IL‐6 is a predominantly anti‐inflammatory cytokine and appears to be protective in heat stress.^42^ Conversely, administration of steroid reduced serum levels of the pro‐inflammatory mediators IL‐1ß,^53^, ^54^ IL‐10,^54^ and TNF‐α^54^; the exception was the low‐dose study by Yang,^55^ where the reduction in TNF‐α was only observed with the combination of mannitol and dexamethasone, but not with either agent alone. However, results in the latter nonsignificant study are recorded after 4 mg kg^−1^, compared with the higher dose of 8 mg kg^−1^ in the study where a difference was seen.

#### 3.2.4 Hepatic, renal, and coagulation system

Two studies detailed the effect of steroid administration on biomarkers of renal and hepatic function, and on clotting factors. The first^54^ showed a statistically significant improvement in biomarkers in all three organ systems after steroid treatment compared with the heated control group; the second study conversely showed a deterioration in markers of liver and renal function and coagulopathy, some reaching statistical significance.^56^

## 4 DISCUSSION

Administration of corticosteroids improved survival time and organ dysfunction due to heat stress, and a reduction in endotoxin and pro‐inflammatory mediators in 80% of the studies included in the review. In addition, administration of another anti‐inflammatory agent in combination with a corticosteroid in one study improved outcomes more than with one agent alone.^55^ The deterioration in the condition of the rats following adrenalectomy with improvement after the addition of dexamethasone^53^ further suggest that moderating the inflammatory response in heat stress is of benefit. The results of the final study suggest a worsening in mortality and markers of organ failure, at variance with the other studies. There are methodological and reporting differences between the studies, which make combining the conclusions less robust. There are differences in the outcome measure for mortality. Some studies used the time at which death occurred, others the time after onset of heat stress. The definition of heatstroke also differed across the studies: in one study, heatstroke was defined as the time that systolic blood pressure fell below 90 mmHg,^56^ and in others, the time at which MAP and CBF fell.^53^, ^54^, ^55^ However, heatstroke in humans may be present without changes in blood pressure.

No human studies were identified for the review, and the application of the studies to clinical practice in humans is uncertain. The two animal models used in the studies, rats and primates, have been shown to show similar inflammatory, metabolic, and cardiovascular features to humans when subjected to heat stress.^57^, ^58^ However, the possibility of interspecies variation in the stress response and pharmacodynamics of corticosteroids cannot be eliminated; in addition, the current working definition of exertional heatstroke requires a core temperature of 40.5°C and central neurological dysfunction,^10^ which would not be possible to discern in an anesthetized animal model. Furthermore, anesthesia has more recently been observed to affect the inflammatory response.^59^, ^60^ All the subjects were anesthetized in these studies, but the implications are unclear. In three of the studies,^53^, ^54^, ^55^ the subjects were anesthetized using urethane, which is known to cause immunosuppression.^61^ The other two studies^50^, ^56^ used ketamine, which is associated with reduction in the pro‐inflammatory TNF‐α and IL‐1,^62^ higher levels of which are associated with adverse outcomes, but also a reduction of IL‐6^62^; which in turn is associated with improved outcome upon systemic increase.

Further evidence that steroids may be effective in human heat stress come from a number of recently published case reports. In one, persistent cardiovascular failure and high serum cytokine levels, associated with a worse outcome,^39^, ^41^, ^42^, ^43^ improved after the administration of hydrocortisone, and the patient was subsequently discharged home.^63^ Similarly, out of five patients admitted with classical heatstroke, three were treated with blood purification therapy, who subsequently survived, while the two who only received conventional therapy died.^64^ The study authors propose that the improved outcome was due to the removal of pro‐inflammatory cytokines,^64^ suggesting that glucocorticoids may have a similar effect.^47^, ^48^

The administration of steroids before onset of heat stress in three of the studies made these data less relevant to clinical practice. Two of the remaining studies, where steroids were given after onset of the heat stress, showed an improvement in mortality and organ dysfunction, although the effect of steroids administered after the insult was lower.^53^

The optimal dose of corticosteroid from these studies is also uncertain. Two studies^53^, ^54^ showed a dose‐dependent improvement. In the study by Yang^55^ where the effect of dexamethasone on cytokine levels and neuronal damage was not significant, the dose used was 50% of the dose used in the study with similar methodology where significant differences were observed. The optimum glucocorticoid and duration of treatment were not addressed in any of the studies, and remain to be determined. Four studies used dexamethasone, and one used methylprednisolone. Both steroids have predominantly glucocorticoid activity. Dexamethasone is long acting, with a biological half life between 36 and 72 hours,^65^ with suppression of the hypothalamic‐pituitary axis persisting for up to 2.5 days. However, the duration of the inflammatory response after an episode of heat stress is not known; raised levels of cytokines are still present after 36 hours,^58^ and whether a longer duration of treatment is required has not so far been addressed.

In one study of the effect of dexamethasone on primates showed a nonsignificant worsening in survival and a statistically significant worsening in markers of renal, hepatic, and coagulation function, at variance with all other identified studies. The reason for this discrepancy is unclear. The expected fall in serum levels of cortisol did not occur in the study group compared with the control group, even after 12 hours, which the study authors suggest may be due to steroid resistance or to dysregulation of the hypothalamic‐pituitary‐adrenal axis occurring as a result of the heat stress. The dose used in the study was the lowest of all the included studies and, in particular, lower than that used in the one other primate study, where a reduction in endotoxemia and mortality was observed.^50^ The successful treatment study administered 30 mg kg^−1^ methylprednisolone, equivalent to 5.6 mg kg^−1^ dexamethasone, whereas 2 mg kg^−1^ was used in the study describing the worsening response following steroids. The animals were anesthetized with ketamine, the influence of which on the immune function as discussed above is uncertain.

Treatment of severe hyperthermia irrespective of the cause currently remains limited to rapid cooling and supportive measures in the majority of cases. Development of new treatments to reduce the associated morbidity and mortality is urgently required. Administration of corticosteroids appears promising and warrants further investigation.

Gastrointestinal permeability and a pro‐inflammatory response appear to occur as a consequence of increased thermal load, irrespective of the cause^14^, ^24^, ^25^, ^26^, ^27^, ^39^; whether corticosteroids are efficacious in hyperthermia of any etiology is unclear but would also warrant further investigation. Identifying a particular cause of a raised temperature is often difficult, but may be noninfectious in up to two‐thirds of cases,^66^ suggesting that steroid administration might prove to be beneficial even when the cause cannot be identified.

## 5 CONCLUSION

Heat stress is associated with a profound pro‐inflammatory response. Steroids appear to improve morbidity and mortality in most animal studies, but their relevance to humans in clinical practice is uncertain. Further studies examining dose responses to corticosteroid administration in humans are warranted, notably where delivery occurs after the onset of heat stress.

## CONFLICT OF INTEREST

The authors declare that there is no conflict of interest.

## AUTHOR CONTRIBUTIONS

Both authors were involved in the conceptualization, data collection and analysis, and the writing of the paper. Both authors have seen and approved the final version.

## Data Availability

All available data can be obtained by contacting the corresponding author.

## APPENDIX A PRISMA checklist (taken from reference 51)

**Table:**

Section and topic	Item No	Checklist item
Administrative information		
Title:		
Identification	1a	Identify the report as a protocol of a systematic review
Update	1b	If the protocol is for an update of a previous systematic review, identify as such
Registration	2	If registered, provide the name of the registry (such as PROSPERO) and registration number
Authors:		
Contact	3a	Provide name, institutional affiliation, e‐mail address of all protocol authors; provide physical mailing address of corresponding author
Contributions	3b	Describe contributions of protocol authors and identify the guarantor of the review
Amendments	4	If the protocol represents an amendment of a previously completed or published protocol, identify as such and list changes; otherwise, state plan for documenting important protocol amendments
Support:		
Sources	5a	Indicate sources of financial or other support for the review
Sponsor	5b	Provide name for the review funder and/or sponsor
Role of sponsor or funder	5c	Describe roles of funder(s), sponsor(s), and/or institution(s), if any, in developing the protocol
**Introduction**		
Rationale	6	Describe the rationale for the review in the context of what is already known
Objectives	7	Provide an explicit statement of the question(s) the review will address with reference to participants, interventions, comparators, and outcomes (PICO)
**Methods**		
Eligibility criteria	8	Specify the study characteristics (such as PICO, study design, setting, time frame) and report characteristics (such as years considered, language, publication status) to be used as criteria for eligibility for the review
Information sources	9	Describe all intended information sources (such as electronic databases, contact with study authors, trial registers or other gray literature sources) with planned dates of coverage
Search strategy	10	Present draft of search strategy to be used for at least one electronic database, including planned limits, such that it could be repeated
Study records:		
Data management	11a	Describe the mechanism(s) that will be used to manage records and data throughout the review
Selection process	11b	State the process that will be used for selecting studies (such as two independent reviewers) through each phase of the review (ie, screening, eligibility and inclusion in meta‐analysis)
Data collection process	11c	Describe planned method of extracting data from reports (such as piloting forms, done independently, in duplicate), any processes for obtaining and confirming data from investigators
Data items	12	List and define all variables for which data will be sought (such as PICO items, funding sources), any preplanned data assumptions and simplifications
Outcomes and prioritization	13	List and define all outcomes for which data will be sought, including prioritization of main and additional outcomes, with rationale
Risk of bias in individual studies	14	Describe anticipated methods for assessing risk of bias of individual studies, including whether this will be done at the outcome or study level, or both; state how this information will be used in data synthesis
Data synthesis	15a	Describe criteria under which study data will be quantitatively synthesized
	15b	If data are appropriate for quantitative synthesis, describe planned summary measures, methods of handling data and methods of combining data from studies, including any planned exploration of consistency (such as I^2^, Kendall's τ)
	15c	Describe any proposed additional analyses (such as sensitivity or subgroup analyses, meta‐regression)
	15d	If quantitative synthesis is not appropriate, describe the type of summary planned
Meta‐bias(es)	16	Specify any planned assessment of meta‐bias(es) (such as publication bias across studies, selective reporting within studies)
Confidence in cumulative evidence	17	Describe how the strength of the body of evidence will be assessed (such as GRADE)

## APPENDIX B Search terms

**Table:**

# Database Search	Database	Search term	Number of results
6	EMBASE	exp "HEAT STRESS"/	9843
7	EMBASE	exp "HEAT INJURY"/	7262
8	EMBASE	exp HYPERTHERMIA/	26 550
9	EMBASE	(6 OR 7 OR 8)	41 854
26	EMBASE	(cancer).ti,ab	2 205 759
34	EMBASE	exp STEROID/	1 416 567
35	EMBASE	(9 AND 34)	2058
36	EMBASE	35 NOT 26	1967
42	EMBASE	35 NOT 26 [Clinical trials Clinical Trial OR Randomized Controlled Trial OR Controlled Clinical Trial OR Multicenter Study]	137
18	PubMed	(heat illness).ti,ab	556
20	PubMed	(heat stroke).ti,ab	3292
21	PubMed	(heat stress).ti,ab	54 325
22	PubMed	(hyperthermia).ti,ab	237 457
23	PubMed	(18 OR 20 OR 21 OR 22)	290 007
28	PubMed	(cancer OR tumour OR chemo*).ti,ab	4 353 854
39	PubMed	(steroid*).ti,ab	332 196
40	PubMed	(23 AND 39)	6407
41	PubMed	40 NOT 28	5340
43	PubMed	(18 AND 39)	3
44	PubMed	(20 AND 39)	24
45	PubMed	(21 AND 39)	719
46	PubMed	(22 AND 39)	5717
13	MEDLINE	exp "HEAT STRESS DISORDERS"/	5306
37	MEDLINE	exp STEROIDS/	838 042
38	MEDLINE	13 AND 37	123
